# A general method for parameter estimation in light-response models

**DOI:** 10.1038/srep27905

**Published:** 2016-06-13

**Authors:** Lei Chen, Zhong-Bin Li, Cang Hui, Xiaofei Cheng, Bai-Lian Li, Pei-Jian Shi

**Affiliations:** 1 Graduate School of Environmental Science, Hokkaido University, N19W8, Sapporo 060-0819, Japan; 2 College of Forestry, Sichuan Agricultural University, 211 Huimin Road, 611130 Wenjiang, Sichuan, China; 3 Agricultural and Forestry Bureau in Qingbaijiang District, 159 Huajin Road, Qingbaijiang 610300, Sichuan, China; 4 Centre for Invasion Biology, Department of Mathematical Sciences, Stellenbosch University, Matieland, South Africa; 5 Mathematical and Physical Biosciences, African Institute for Mathematical Sciences, Cape Town, South Africa; 6 Co-Innovation Centre for Sustainable Forestry in Southern China, Bamboo Research Institute, Nanjing Forestry University, 159 Longpan Road, Nanjing 210037, China; 7 Ecological Complexity and Modelling Laboratory, Department of Botany and Plant Sciences, University of California, Riverside, CA 92521-0124, USA

## Abstract

Selecting appropriate initial values is critical for parameter estimation in nonlinear photosynthetic light response models. Failed convergence often occurs due to wrongly selected initial values when using currently available methods, especially the kind of local optimization. There are no reliable methods that can resolve the conundrum of selecting appropriate initial values. After comparing the performance of the Levenberg–Marquardt algorithm and other three algorithms for global optimization, we develop a general method for parameter estimation in four photosynthetic light response models, based on the use of Differential Evolution (DE). The new method was shown to successfully provide good fits (*R*^*2*^ > 0.98) and robust parameter estimates for 42 datasets collected for 21 plant species under the same initial values. It suggests that the DE algorithm can efficiently resolve the issue of hyper initial-value sensitivity when using local optimization methods. Therefore, the DE method can be applied to fit the light-response curves of various species without considering the initial values.

Photosynthesis is one of the most important biological processes involved in plant growth, and the rate of photosynthesis rate can be affected by a list of factors, such as temperature, CO_2_ concentration and light intensity^1^,^2^. In particular, many nonlinear models have been developed for describing the rate of photosynthesis in response to the change of irradiance^3^,^4^,^5^. There are three types of photosynthetic light response models currently available in literature: exponential, rectangular hyperbola and nonrectangular hyperbola.

Exponential Model^6^:


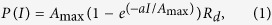


where 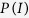 is the net photosynthetic rate, *I* the irradiance, 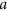 the initial quantum efficiency, *A*_max_ the net light saturated photosynthetic rate, and 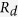 the dark respiration rate.

Rectangular Hyperbolic Model^7^,^8^:


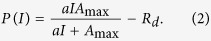


Nonrectangular Hyperbola Model^9^:


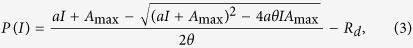


where 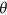 is a curvature parameter.

Modified Rectangular Hyperbola Model^10^,^11^:


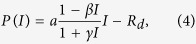


where *β* and γ are constants.

Selecting appropriate initial values is crucial for fitting these nonlinear models and estimating their parameters. For biologically meaningful parameters, using the estimates directly from experiments would be a first try for assigning the initial values. However, for model specific parameters that cannot be measured in experiments, choosing appropriate initial values when fitting the model could become problematic. For example, two of the parameters in Eq. (4) are constants without direct biological meanings. Even for those parameters with direct biological meanings, assigning their initial values in the model fitting could still be time consuming. For instance, all the three parameters in the exponential model (Eq. 1) have sound biological meanings, but only *A*_max_ can be directly measured; the other two parameters, *a* and *R*_*d*_, require additional procedures to derive.

There are many statistical software packages currently available for fitting nonlinear models; to list a few, SAS, R, GenStat, MatLab, Sigmaplot, and SPSS. The built-in functionality to fit nonlinear models in these software packages normally implement algorithms of local optimization for regression and parameter estimation, such as the Gauss-Newton, Levenberg-Marquardt and Nelder-Mead algorithms. As local optimization methods are highly sensitive to the setting of initial values, failed convergence often occurs due to wrongly assigned initial values. However, there are no reliable methods available for selecting initial values^5^.

To develop effective methods for resolving the problem of assigning appropriate initial values when using these nonlinear models, we need to go beyond local optimization. Genetic algorithms and simulated annealing algorithms have been widely applied to search global optimal solutions in multi-dimensional space^12^,^13^,^14^,^15^,^16^,^17^. In particular, recent comparisons have suggested that the Differential Evolution (DE) algorithm performs better than other global optimization methods such as the genetic algorithm and simulated annealing^18^,^19^. There are four main steps for implementing the DE algorithms: initialization, mutation, crossover and selection, which is also capable of dealing with non-differentiable problems^18^. Compared to local optimization methods, global optimization algorithms are less sensitive to initial values^20^,^21^. However, the application of global optimization algorithms to parameter estimation in photosynthetic light-response models is lacking.

The objectives of this study are to: (1) test the performance of the Levenberg-Marquardt (LM) algorithm, a local optimization method, and three global optimization algorithms: Genetic Algorithm (GA), Generalized Simulated Annealing (GSA) and the Differential Evolution (DE) algorithm; (2) develop a reliable method for fitting photosynthetic light-response models using the global optimization algorithm and simultaneously solve the issue of hyper initial-value sensitivity in local optimization algorithms.

## Results and Discussion

### Model comparison

According to calculated values of AIC (Tables 1 and 2), the modified rectangular and the nonrectangular hyperbolic models performed better than the exponential and rectangular hyperbolic models. The nonrectangular model is more flexible for its additional curvature parameter (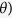; when *θ* = 0, it becomes the rectangular model^5^,^22^. For example, the estimates of 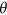 for the photosynthetic datasets of *Brassica rapa* var. *chinensis* (L.) Kitam., *Camellia sinensis* (L.) Kuntze and *Impatiens balsamina* L. were all close to zero, the other three parameters of the nonrectangular model were also equal to those estimated from the rectangular model (Supplementary Tables S1 and S2).

### Comparison of local and global optimization algorithms

**Figure 1:**
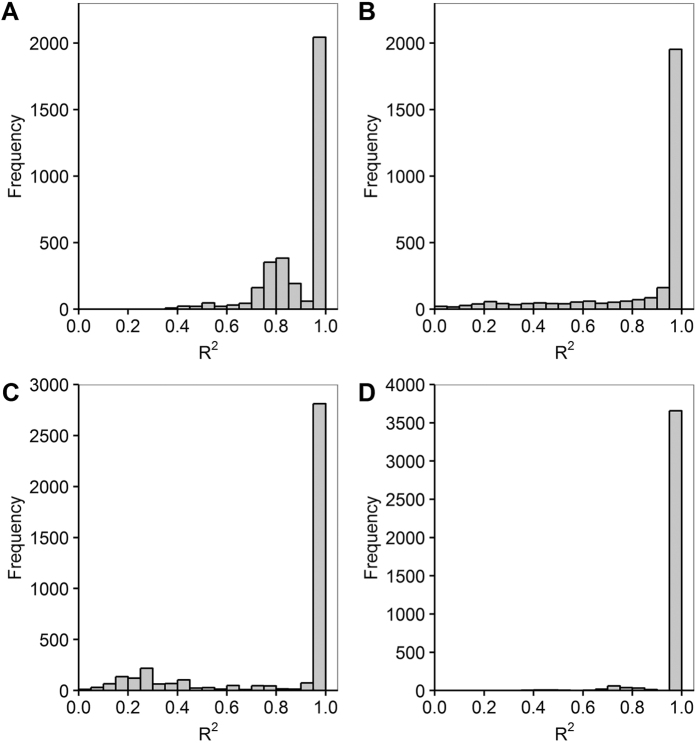
Distribution of *R*^*2*^ when using the Levenberg–Marquardt algorithm with successful convergence of the modified rectangular (**A**), nonrectangular (**B**), rectangular (**C**) and exponential (**D**) models.

**Figure 2:**
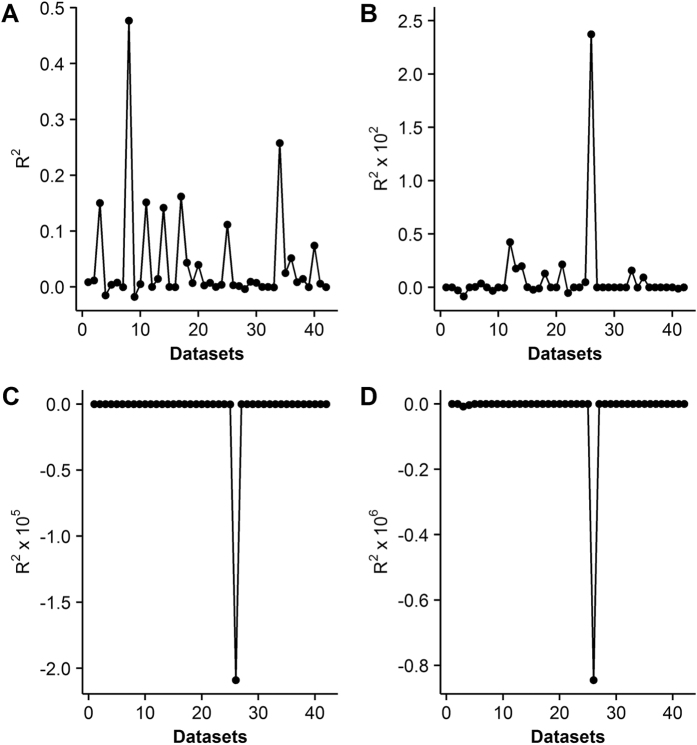
Difference of *R*^*2*^ when using the Differential Evolution and Generalized Simulated Annealing algorithm for the modified rectangular (**A**), nonrectangular (**B**), rectangular (**C**) and exponential (**D**) models.

**Figure 3:**
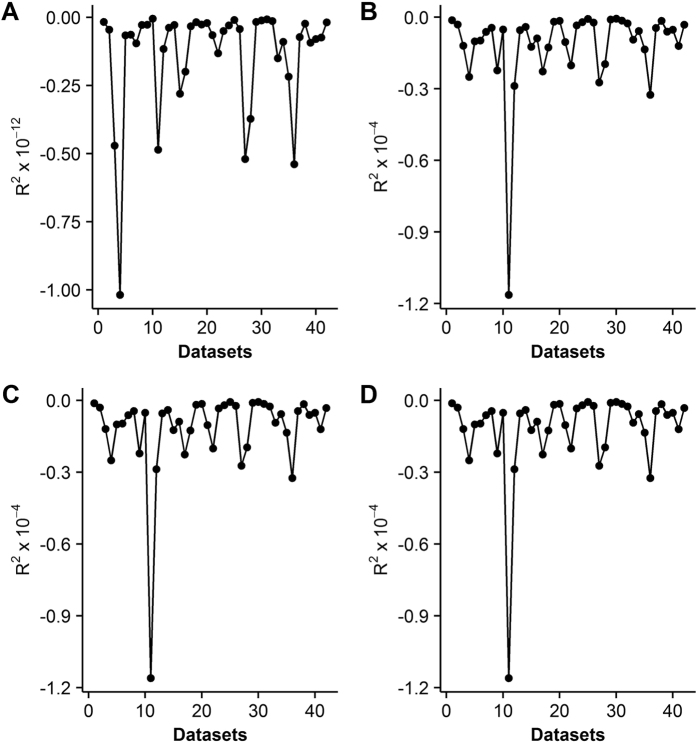
Values of *R*^*2*^ when using the Genetic Algorithm for the modified rectangular (**A**), nonrectangular (**B**), rectangular (**C**) and exponential (**D**) models.

**Figure 4:**
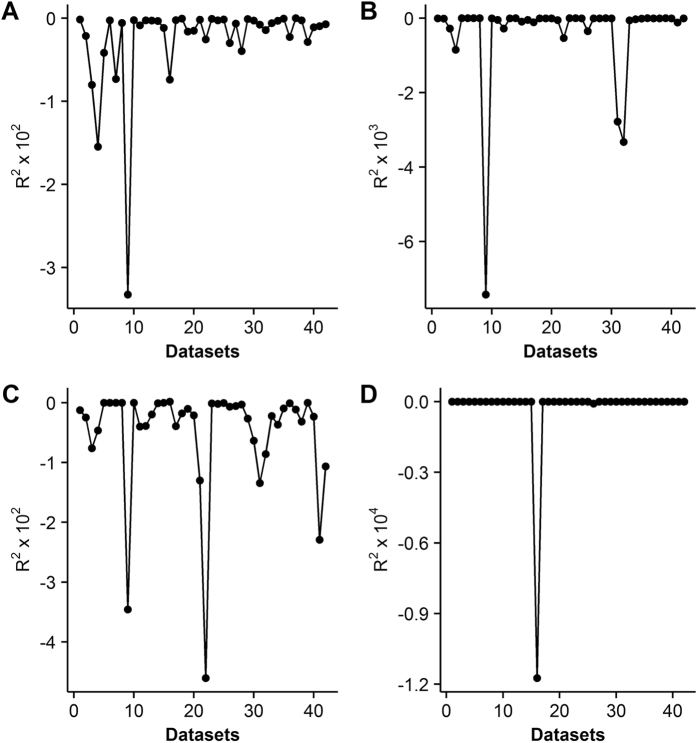
Difference of *R*^*2*^ when using the Differential Evolution and the best optimum from the Levenberg–Marquardt algorithm for the modified rectangular (**A**), nonrectangular (**B**), rectangular (**C**) and exponential (**D**) models.

**Figure 5:**
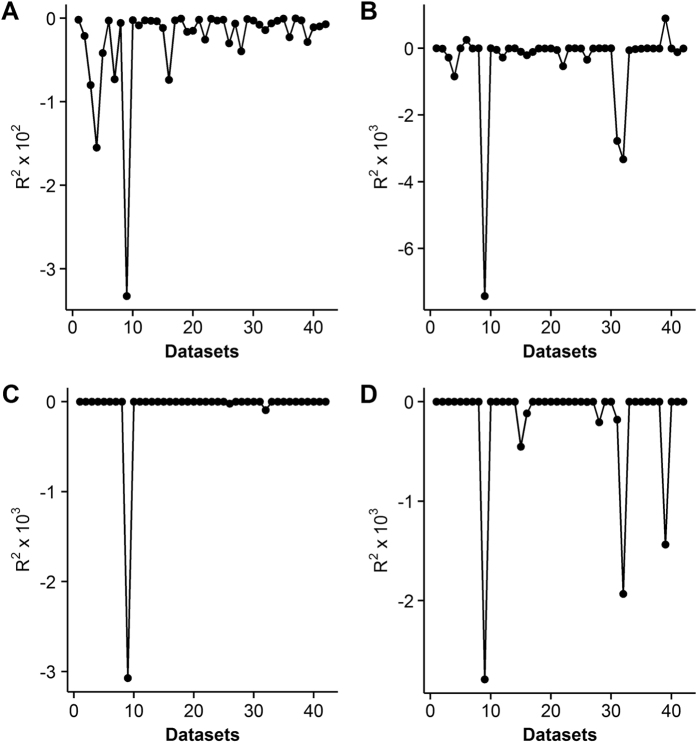
Difference of *R*^*2*^ when using the Differential Evolution, combined with the Levenberg–Marquardt algorithm for the modified rectangular (**A**), nonrectangular (**B**), rectangular (**C**) and exponential (**D**) models.

Not surprisingly, failed convergence often occurred from wrongly assigned initial values when only using the LM algorithm, due to its hyper sensitivity to initial values (Table 3). In particular, the nonrectangular hyperbolic model failed to converge for 1260 times due to inappropriate initial values in the simulation (Table 3, Supplementary Table S3). There were also many compromised fits with *R*^*2*^ < 0.90 even when the LM algorithm converged successfully (Fig. 1). By contrast, the DE algorithm was less sensitive to initial values, which provided good fits with *R*^*2*^ approximately 0.98 for all the 42 datasets of 21 species (Tables 1 and 2) when using the same initial values for each photosynthetic model, as listed in Table 4. The DE also performed the best among the three global optimization methods, followed by the GSA (Fig. 2). In comparison, parameter estimates from the GA were not reliable with even negative *R*^*2*^(Fig. 3). The speed of convergence for the GA algorithm was also slower than that of the DE and GSA algorithms. Although the best optimum solutions of the 100 simulation tests from the LM were similar to those from the DE (Fig. 4), however, it was not recommended to only use the LM as the large amount of failures occurred due to wrongly sampled initial values (Table 3). After setting the initial values to the parameter estimates from the DE algorithm, the value of *R*^2^ estimated from the LM algorithm only slightly increased (<0.05) for the rectangular and modified rectangular models; however, the values of *R*^*2*^ almost remained the same for the exponential and nonrectangular models (Fig. 5). Although local optimization methods are sensitive to the input of initial values, local optimization algorithms are generally faster and more accurate than global optimization method in a local space^20^,^23^,^24^. In addition, there was no failures of convergence when the parameters of rectangular and modified rectangular models obtained from the DE algorithm were set as the initial values for running the LM algorithm. Therefore, we recommend to combine the DE and LM algorithms for estimating parameters of the rectangular and modified rectangular models if higher accuracy is needed, instead of increasing the number of iterations when running the DE algorithm.

In conclusion, the application of the DE algorithm can not only effectively resolve the issue of hyper sensitivity of initial values in local optimization but also provide good fits for the photosynthetic models. The lower and upper bounds of initial values (Table 4) for each photosynthetic light-response model can be regarded as a standard setting when using the DE algorithm. In addition, the estimated parameters (Supplementary Tables S1 and S2) of the mainstream photosynthetic models for both herbaceous and woody plant species can be used as a reference for selecting initial values when using the traditional local optimization method.

## Methods

A total of 42 datasets from 21 species (Supplementary Table S4), including both herbaceous and woody plants, were used for evaluating the performance of three global optimization algorithms: Genetic Algorithm (GA), Generalized Simulated Annealing (GSA) and the Differential Evolution (DE) algorithms, when fitting the photosynthetic light response models. For comparison, the Levenberg-Marquardt (LM) algorithm, a derivative based local optimization method, was also applied to estimate parameters of the photosynthetic models. The raw data of species 2, 5–9, 14–17, 20 and 22 (see Supplementary Table S4) were extracted from figures in publications^25^,^26^,^27^,^28^,^29^,^30^,^31^,^32^,^33^,^34^,^35^,^36^ using the GetData Graph Digitizer 2.26 (http://getdata-graph-digitizer.com). After 500 iterations, the estimates from the three optimization methods were used to evaluate their performance. To avoid biasing the results, we used the same lower and upper bounds for parameter estimation when using the three global optimization (Table 4). For each dataset, 100 random values were drew from a uniform distribution with the same lower and upper bounds for the global optimization algorithms as initial values for testing the LM algorithm. A total of 4200 simulations were repeated for each photosynthetic model. Parameters of all the photosynthetic light-response models were estimated by the LM, GA, GSA and DE algorithms, implemented in the minpack.lm, GA, GenSA, and DEoptim packages of R^37^. The coefficient of determination 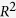 and Akaike’s information criterion (AIC)^38^ were used as the goodness-of-fit index and for model comparison, respectively.

## Additional Information

**How to cite this article**: Chen, L. *et al*. A general method for parameter estimation in light-response models. *Sci. Rep.* **6**, 27905; doi: 10.1038/srep27905 (2016).

## Supplementary Material

Supplementary Information

Supplementary Dataset

## Figures and Tables

**Table 1:** Akaike’s information criterion (AIC) and *R*^*2*^ values of each model using the Differential Evolution algorithm.

Species	Exponential		Rectangular		Nonrectangular		Modified Rectangular	
	R square	AIC	R square	AIC	R square	AIC	R square	AIC
1	0.999	−21.42	0.998	−13.58	0.999	−23.14	0.999	−15.01
	0.998	−25.65	0.995	−14.58	0.998	−21.34	0.998	−22.42
2	0.987	−17.50	0.974	−9.90	0.98	−11.25	0.990	−18.15
	0.961	−14.18	0.953	−12.15	0.96	−11.12	0.970	−14.98
3	0.979	−10.42	0.995	−25.89	0.995	−23.89	0.994	−22.09
	0.991	−19.81	0.998	−34.71	0.998	−32.71	0.997	−29.49
4	0.965	0.28	0.985	−9.69	0.985	−7.69	0.985	−7.55
	0.988	−6.87	0.994	−14.62	0.994	−12.62	0.994	−12.10
5	0.978	−18.15	0.960	−11.71	0.99	−21.75	0.975	−14.85
	0.994	−18.38	0.999	−37.09	1.00	−35.06	0.999	−37.72
6	0.990	−36.34	0.992	−37.97	1.00	−41.01	0.989	−33.37
	0.997	−34.44	0.992	−25.48	1.00	−29.27	0.998	−35.79
7	0.999	−25.26	0.998	−16.01	1.00	−32.13	0.999	−22.95
	0.997	−11.62	0.998	−13.86	1.00	−12.09	0.998	−13.15
8	0.986	−20.87	0.996	−38.73	1.00	−36.62	0.996	−35.76
	0.973	−7.89	0.984	−14.25	0.98	−12.19	0.982	−10.89
9	0.996	−26.03	0.990	−18.43	0.99	−20.07	0.999	−37.43
	0.998	−28.81	0.998	−25.38	1.00	−34.55	1.000	−38.14
10	0.999	−23.71	0.998	−17.03	0.999	−22.01	0.999	−21.12
	0.995	−5.62	0.994	−2.39	0.996	−5.40	0.995	−3.93

**Table 2:** Akaike’s information criterion (AIC) and *R* ^*2*^ values of each model using the Differential Evolution algorithm.

Species	Exponential		Rectangular		Nonrectangular		Modified Rectangular	
	R square	AIC	R square	AIC	R square	AIC	R square	AIC
11	0.991	−19.48	0.975	−8.64	0.988	−14.65	0.993	−21.20
	0.958	−10.46	0.919	−3.32	0.965	−10.52	0.984	−19.03
12	0.992	−8.97	0.996	−16.51	0.996	−14.83	0.995	−13.51
	0.998	−17.39	0.999	−25.17	0.999	−25.08	0.998	−20.70
13	0.999	−10.09	1.000	−16.75	1.00	−15.45	1.000	−14.75
	0.999	−19.33	0.999	−14.16	1.00	−14.69	0.998	−7.84
14	0.997	−33.31	0.999	−41.85	1.00	−44.65	0.992	−23.31
	0.992	−22.77	0.994	−24.92	0.99	−23.36	0.996	−26.88
15	0.998	−4.91	0.996	1.26	1.00	−5.05	0.998	−1.05
	0.998	1.95	0.993	9.79	1.00	−1.34	0.996	6.48
16	0.995	−3.05	0.984	10.86	0.99	0.32	0.997	−7.49
	0.995	−11.99	0.988	−0.58	0.99	−5.62	0.994	−7.58
17	0.995	−28.86	0.994	−26.81	0.996	−30.64	0.995	−27.36
	0.997	−30.19	0.995	−22.64	0.999	−37.67	0.997	−27.04
18	0.994	−30.15	0.994	−31.26	0.995	−31.42	0.994	−28.23
	0.982	−28.23	0.984	−29.42	0.984	−27.41	0.982	−26.21
19	0.996	−18.87	0.998	−27.78	1.00	−36.33	0.999	−28.39
	1.000	−32.57	0.996	−6.78	1.00	−23.79	1.000	−28.34
20	0.987	−10.90	0.996	−24.77	0.996	−22.77	0.993	−17.47
	0.998	−31.17	0.997	−27.28	0.999	−45.01	0.998	−28.24
21	0.991	−14.11	0.968	−3.82	0.99	−11.83	0.985	−7.63
	0.997	−10.88	0.989	−0.85	1.00	−21.03	0.995	−5.20

**Table 3:** Performance of the Levenberg-Marquardt in the simulation test.

Models	Times of failed convergence
Exponential Model	358
Rectangular Hyperbolic Model	251
Nonrectangular Hyperbola Model	1260
Modified Rectangular Hyperbola Model	820

**Table 4:** Lower and upper bounds of parameters for each model optimized by the Genetic Algorithm, Generalized Simulated Annealing and the Differential Evolution algorithms.

Models	Parameters	Lower bound	Upper bound
Exponential Model	*a*	0	1
	*Amax*	0	100
	*Rd*	0	100
Rectangular Hyperbolic Model	*a*	0	1
	*Amax*	0	100
	*Rd*	0	100
Nonrectangular Hyperbola Model	*θ*	0	1
	*a*	0	1
	*Amax*	0	100
	*Rd*	0	100
Modified Rectangular Hyperbola Model	*a*	0	1
	*β*	0	1
	*γ*	0	1
	*Rd*	0	100

The maximum number of iteration is 500.

## References

[b1] Farquhar G. D. & Sharkey T. D. Stomatal conductance and photosynthesis. Annu. Rev. Plant Physiol. 33, 317–345 (1982).

[b2] Farquhar G. D., Ehleringer J. R. & Hubick K. T. Carbon isotope discrimination and photosynthesis. Annu. Rev. Plant Biol. 40, 503–537 (1989).

[b3] Jassby A. D. & Platt T. Mathematical formulation of the relationship between photosynthesis and light for phytoplankton. Limnol. Oceanogr. 21, 540–547 (1976).

[b4] Lieth J. & Reynolds J. The nonrectangular hyperbola as a photosynthetic light response model: geometrical interpretation and estimation of the parameter. Photosynthetica 21, 363–365 (1987).

[b5] Archontoulis S. V. & Miguez F. E. Nonlinear regression models and applications in agricultural research. Agron. J. 107, 786–798 (2015).

[b6] Bassman J. H. & Zwier J. C. Gas exchange characteristics of Populus trichocarpa, Populus deltoides and Populus trichocarpa× P. deltoides clones. Tree Physiol. 8, 145–159 (1991). 14972886 10.1093/treephys/8.2.145

[b7] Kyei-Boahen S., Lada R., Astatkie T., Gordon R. & Caldwell C. Photosynthetic response of carrots to varying irradiances. Photosynthetica 41, 301–305 (2003).

[b8] Thornley J. Dynamic model of leaf photosynthesis with acclimation to light and nitrogen. Ann. Bot. 81, 421–430 (1998).

[b9] Marshall B. & Biscoe P. A model for C3 leaves describing the dependence of net photosynthesis on irradiance. J. Exp. Bot. 31, 29–39 (1980).

[b10] Ye Z.-P. A new model for relationship between irradiance and the rate of photosynthesis in Oryza sativa. Photosynthetica 45, 637–640 (2007).

[b11] Ye Z.-P. & Yu Q. A coupled model of stomatal conductance and photosynthesis for winter wheat. Photosynthetica 46, 637–640 (2008).

[b12] Tsallis C. & Stariolo D. A. Generalized simulated annealing. Physica A 233, 395–406 (1996).

[b13] Xiang Y., Gubian S., Suomela B. & Hoeng J. Generalized simulated annealing for global optimization: the GenSA Package. R Journal 5, 13–28 (2013).

[b14] Hartmann S. A competitive genetic algorithm for resource‐constrained project scheduling. Nav. Res. Logist. 45, 733–750 (1998).

[b15] Romeijn H. E. & Smith R. L. Simulated annealing for constrained global optimization. J. Global Optim. 5, 101–126 (1994).

[b16] Mendi F., Başkal T., Boran K. & Boran F. E. Optimization of module, shaft diameter and rolling bearing for spur gear through genetic algorithm. Expert. Syst. Appl. 37, 8058–8064 (2010).

[b17] Song S. & Singh V. P. Frequency analysis of droughts using the Plackett copula and parameter estimation by genetic algorithm. Stoch. Environ. Res. Risk Assess. 24, 783–805 (2010).

[b18] Storn R. & Price K. Differential evolution–a simple and efficient heuristic for global optimization over continuous spaces. J. Global Optim. 11, 341–359 (1997).

[b19] Vesterstrøm J. & Thomsen R. A comparative study of differential evolution, particle swarm optimization, and evolutionary algorithms on numerical benchmark problems in Evolutionary Computation, 2004. CEC2004. Congress on, Vol. 2, 1980-1987 (IEEE, 2004).

[b20] Ge J.-X., Chou S.-C. & Gao X.-S. Geometric constraint satisfaction using optimization methods. Comput.-Aided Des. 31, 867–879 (1999).

[b21] Brun F., Wallach D., Makowski D. & Jones J. W. Working with dynamic crop models: evaluation, analysis, parameterization, and applications (Elsevier 2006).

[b22] Ögren E. & Evans J. Photosynthetic light-response curves. Planta 189, 182–190 (1993).

[b23] Wang L., Gong M., Gong M. & Yang R. How far can we go with local optimization in real-time stereo matching in 3D Data Processing, Visualization, and Transmission. Third International Symposium on. Vol. 0 129–136 (IEEE, 2006).

[b24] Moeslund T. B., Hilton A., Krüger V. & Sigal L. Visual analysis of humans. (Springer 2011).

[b25] Aleric K. M. & Kirkman L. K. Growth and photosynthetic responses of the federally endangered shrub, Lindera melissifolia (Lauraceae), to varied light environments. Am. J. Bot. 92, 682–689 (2005). 21652446 10.3732/ajb.92.4.682

[b26] Rzigui T. . Light acclimation of leaf gas exchange in two Tunisian cork oak populations from contrasting environmental conditions. IFOREST 8, 700–706 (2015).

[b27] Samuelson L. J. & Stokes T. A. Leaf physiological and morphological responses to shade in grass-stage seedlings and young trees of longleaf pine. Forests 3, 684–699 (2012).

[b28] Zhao D., Glaz B. & Comstock J. C. Sugarcane leaf photosynthesis and growth characters during development of water-deficit stress. Crop Sci. 53, 1066–1075 (2013).

[b29] Zhang L. & Xing D. Rapid determination of the damage to photosynthesis caused by salt and osmotic stresses using delayed fluorescence of chloroplasts. Photochem. Photobiol. Sci. 7, 352–360 (2008). 18389153 10.1039/b714209a

[b30] Nabity P., Heng-Moss T. & Higley L. G. Effects of insect herbivory on physiological and biochemical (oxidative enzyme) responses of the halophyte Atriplex subspicata (Chenopodiaceae). Environ. Entomol. 35, 1677–1689 (2006).

[b31] Hüner N. P. . Potential for increased photosynthetic performance and crop productivity in response to climate change: role of CBFs and gibberellic acid. Front. Chem. 2, 18 (2014). 24860799 10.3389/fchem.2014.00018 PMC4029004

[b32] Atwell B. J., Kriedemann P. E. & Turnbull C. G. Plants in action: adaptation in nature, performance in cultivation (Macmillan Education AU 1999).

[b33] Jorquera-Fontena E., Alberdi M. & Franck N. Pruning severity affects yield, fruit load and fruit and leaf traits of’Brigitta’blueberry. J. Soil Sci. Plant Nutr. 14, 855–868 (2014).

[b34] Yu X., Hyldgaard B., Rosenqvist E., Ottosen C.-O. & Chen J. Interspecific hybridization in Cucumis leads to the divergence of phenotypes in response to low light and extended photoperiods. Front. Plant Sci. 6 (2015), 10.3389/fpls.2015.00802. PMC4591486 26483817

[b35] Huang W., Zhang S.-B. & Hu H. Sun leaves up-regulate the photorespiratory pathway to maintain a high rate of CO2 assimilation in tobacco. Frontiers in plant science 5 (2014), 10.3389/fpls.2014.00688. PMC4253947 25520735

[b36] Prieto J. A., Giorgi E. G. & Peña J. P. Modelling photosynthetic-light response on Syrah leaves with different exposure. VITIS-J.Grap. Res. 49, 145 (2015).

[b37] R Core Team (2015). R: A language and environment for statistical computing. R Foundation for Statistical Computing, Vienna, Austria. Available at: https://www.R-project.org/. (Date of access: 11/27/2015).

[b38] Posada D. & Buckley T. R. Model selection and model averaging in phylogenetics: advantages of Akaike information criterion and Bayesian approaches over likelihood ratio tests. Syst. Biol. 53, 793–808 (2004). 15545256 10.1080/10635150490522304

