# Supplementary material for: A general method for parameter estimation in light-response models
Source: Sci Rep. 2016 Jun 13;6:27905. doi: 10.1038/srep27905 (PMC4904205; doi:10.1038/srep27905)
Supplement: Supplementary Information [file srep27905-s1.pdf]

Supplementary information for

A general method for parameter estimation in light-response models

Lei Chen[1], Zhong-Bin Li[2,3], Cang Hui[4,5], Xiaofei Cheng[6], Bai-Lian Li[7] and Pei-Jian Shi[*,6]

[1] Graduate School of Environmental Science, Hokkaido University, N19W8, Sapporo 060-0819, Japan

[2] College of Forestry, Sichuan Agricultural University, 211 Huimin Road, 611130 Wenjiang, Sichuan, China

[3] Agricultural and Forestry Bureau in Qingbaijiang District, 159 Huajin Road, Qingbaijiang 610300, Sichuan, China

[4] Centre for Invasion Biology, Department of Mathematical Sciences, Stellenbosch University, Matieland, South Africa

[5] Mathematical and Physical Biosciences, African Institute for Mathematical Sciences, Cape Town, South Africa

[6] Co-Innovation Centre for Sustainable Forestry in Southern China, Bamboo Research Institute, Nanjing Forestry University, 159 Longpan Road, Nanjing 210037, China

[7] Ecological Complexity and Modelling Laboratory, Department of Botany and Plant Sciences, University of California, Riverside, CA 92521-0124, USA

Table S1−1. Estimated parameter values of the photosynthetic light-response models using the Differential Evolution algorithm.

Species	Exponential			Rectangular			Nonrectangular				Modified Rectangular			
	a	Amax	Rd	a	Amax	Rd	a	Amax	θ	Rd	a	β	γ	Rd
1	0.05	27.38	1.83	0.07	35.45	2.33	0.05	31.06	0.58	1.86	0.07	2.99E-05	1.79E-03	2.37
	0.05	16.60	1.57	0.07	20.26	2.08	0.05	18.11	0.63	1.59	0.06	8.22E-05	2.42E-03	1.72
2	0.04	9.64	3.91	0.06	11.02	4.13	0.03	10.18	0.71	3.80	0.06	7.27E-05	4.44E-03	4.40
	0.04	6.74	4.02	0.07	7.40	4.14	0.04	7.28	0.66	4.18	0.05	7.74E-05	5.67E-03	3.57
3	0.04	9.03	1.59	0.07	10.88	2.07	0.07	10.88	1.70E-06	2.07	0.07	1.15E-06	6.72E-03	2.26
	0.04	9.24	1.51	0.05	11.30	1.88	0.05	11.30	6.86E-05	1.88	0.06	2.64E-06	4.80E-03	2.11
4	0.05	11.48	1.38	0.08	13.64	1.93	0.08	13.64	9.36E-06	1.93	0.08	2.61E-08	5.62E-03	1.78
	0.05	13.98	1.33	0.07	17.35	1.77	0.06	17.28	0.03	1.76	0.06	3.40E-06	3.61E-03	1.83
5	0.03	7.72	0.00	0.04	9.81	0.00	0.02	7.70	0.94	0.01	0.04	2.34E-04	2.87E-03	0.01
	0.09	13.67	0.79	0.14	16.59	1.39	0.14	16.59	0.01	1.38	0.14	3.51E-05	8.08E-03	1.38
6	0.03	2.74	0.06	0.05	3.12	0.13	0.03	2.94	0.56	0.08	0.06	4.81E-06	1.84E-02	0.23
	0.05	5.55	0.34	0.09	6.36	0.54	0.05	5.93	0.66	0.39	0.07	1.11E-04	9.98E-03	0.49
7	0.05	10.83	1.00	0.07	12.95	1.30	0.05	11.87	0.60	1.05	0.06	1.17E-04	3.79E-03	1.20
	0.05	12.86	1.22	0.08	15.52	1.69	0.07	15.23	0.16	1.61	0.07	2.37E-05	4.54E-03	1.68
8	0.03	8.66	0.00	0.05	10.23	0.32	0.05	10.20	0.00	0.34	0.05	1.09E-08	5.07E-03	0.25
	0.02	10.19	0.00	0.04	12.62	0.52	0.04	12.56	0.00	0.44	0.04	3.70E-08	3.27E-03	0.95
9	0.07	5.95	2.67	0.14	6.88	3.05	0.07	6.23	0.70	2.67	0.10	1.85E-04	1.28E-02	2.78
	0.06	7.79	1.77	0.10	9.13	1.99	0.07	8.56	0.56	1.86	0.09	1.14E-04	8.87E-03	1.96
10	0.05	21.85	1.53	0.07	27.53	2.09	0.05	24.88	0.53	1.64	0.06	1.26E-04	1.54E-03	1.74
	0.06	24.41	2.40	0.08	30.81	2.95	0.06	27.05	0.63	2.43	0.07	1.04E-04	1.61E-03	2.59

Table S1−2. Estimated parameter values of the photosynthetic light-response models using the Differential Evolution algorithm.

Species	Exponential			Rectangular			Nonrectangular				Modified Rectangular			
	a	Amax	Rd	a	Amax	Rd	a	Amax	θ	Rd	a	β	γ	Rd
11	0.06	9.31	0.43	0.08	10.86	0.76	0.04	9.19	0.90	0.14	0.06	1.94E-04	4.21E-03	0.42
	0.05	6.55	0.69	0.07	7.44	0.88	0.02	5.98	1.00	0.18	0.04	2.75E-04	3.79E-03	0.37
12	0.05	15.71	0.42	0.06	19.38	0.90	0.06	18.86	0.19	0.81	0.07	8.91E-06	3.37E-03	1.03
	0.05	20.69	0.79	0.07	25.90	1.39	0.06	24.82	0.27	1.21	0.07	7.77E-05	2.14E-03	1.35
13	0.07	54.08	3.87	0.09	74.41	5.43	0.08	71.70	0.16	5.09	0.09	3.13E-06	1.20E-03	5.49
	0.05	31.43	3.52	0.09	42.30	6.64	0.07	39.54	0.32	5.38	0.07	2.53E-09	1.80E-03	4.74
14	0.02	4.93	0.10	0.03	5.78	0.26	0.03	5.56	0.36	0.20	0.05	1.54E-08	7.81E-03	0.57
	0.02	5.79	0.00	0.03	6.74	0.10	0.03	6.54	0.29	0.06	0.03	8.28E-05	3.45E-03	0.05
15	0.07	30.36	2.31	0.09	38.29	2.86	0.07	33.45	0.65	2.31	0.08	9.48E-05	1.69E-03	2.85
	0.10	40.56	3.08	0.12	50.87	3.60	0.08	42.65	0.79	2.73	0.10	1.12E-04	1.48E-03	2.97
16	0.10	25.59	0.00	0.13	31.06	0.26	0.08	26.46	0.86	0.00	0.11	2.20E-04	2.19E-03	0.15
	0.09	19.21	0.00	0.13	22.45	0.00	0.09	20.58	0.69	0.00	0.12	1.20E-04	4.25E-03	0.18
17	0.05	9.33	2.22	0.07	10.90	2.57	0.05	10.09	0.57	2.29	0.06	6.95E-05	4.71E-03	2.29
	0.05	11.86	1.92	0.06	14.18	2.26	0.04	12.80	0.65	1.96	0.06	7.58E-05	3.57E-03	2.25
18	0.03	7.58	1.00	0.04	9.09	1.27	0.03	8.53	0.48	1.13	0.04	3.07E-11	4.96E-03	1.38
	0.02	4.79	0.93	0.03	5.74	1.13	0.03	5.66	0.07	1.08	0.02	1.18E-04	2.71E-03	0.98
19	0.06	13.37	0.81	0.08	15.82	1.28	0.06	14.97	0.45	1.03	0.08	3.72E-05	4.55E-03	1.20
	0.08	23.94	2.31	0.11	28.93	2.98	0.07	25.78	0.69	2.27	0.09	1.11E-04	2.30E-03	2.40
20	0.03	11.81	0.00	0.03	14.80	0.04	0.03	14.80	1.40E-07	0.04	0.03	4.16E-05	1.96E-03	0.08
	0.04	12.58	0.43	0.06	15.16	0.81	0.04	13.75	0.61	0.49	0.06	2.83E-05	3.91E-03	0.77
21	0.07	8.51	0.64	0.10	9.61	0.74	0.04	8.28	0.94	0.33	0.08	1.63E-04	6.80E-03	0.78
	0.05	16.68	0.40	0.07	21.11	0.63	0.04	16.87	0.86	0.11	0.06	1.32E-04	2.11E-03	0.51

Table S2−1. Estimated parameter values of the photosynthetic light-response models using both the Differential Evolution and the Nelder-Mead algorithms.

Species	Exponential			Rectangular			Nonrectangular				Modified Rectangular			
	a	Amax	Rd	a	Amax	Rd	a	Amax	θ	Rd	a	β	γ	Rd
1	0.055	27.38	1.83	0.069	35.45	2.33	0.05	31.14	0.57	1.87	0.061	8.37E-05	1.37E-03	2.08
	0.052	16.60	1.57	0.072	20.26	2.08	0.05	18.08	0.65	1.58	0.060	1.04E-04	2.33E-03	1.78
2	0.04	9.64	3.91	0.06	11.02	4.13	0.04	10.18	0.75	3.96	0.04	1.26E-04	2.90E-03	3.97
	0.04	6.74	4.02	0.07	7.40	4.14	0.04	7.10	0.73	4.10	0.04	1.29E-04	4.41E-03	4.04
3	0.041	9.03	1.59	0.065	10.88	2.07	0.13	12.30	-2.53	2.40	0.084	-1.42E-04	9.16E-03	2.30
	0.035	9.24	1.51	0.051	11.30	1.88	0.06	11.95	-0.59	2.01	0.053	-3.31E-05	4.97E-03	1.93
4	0.053	11.48	1.38	0.081	13.64	1.93	0.15	15.11	-2.17	2.28	0.077	-8.45E-05	6.48E-03	1.63
	0.048	13.98	1.33	0.065	17.35	1.77	0.06	17.28	0.03	1.76	0.069	-4.54E-05	4.28E-03	1.85
5	0.03	7.43	-0.35	0.04	9.57	-0.38	0.02	7.00	0.95	-0.57	0.03	4.83E-04	1.05E-03	-0.48
	0.09	13.67	0.79	0.14	16.59	1.39	0.14	16.55	0.02	1.38	0.13	5.85E-05	7.56E-03	1.30
6	0.03	2.74	0.06	0.05	3.12	0.13	0.03	2.96	0.61	0.10	0.04	6.26E-05	1.29E-02	0.12
	0.05	5.55	0.34	0.09	6.36	0.54	0.06	5.97	0.64	0.41	0.07	1.10E-04	1.02E-02	0.47
7	0.05	10.83	1.00	0.07	12.95	1.30	0.05	11.87	0.60	1.05	0.06	9.71E-05	3.92E-03	1.17
	0.05	12.86	1.22	0.08	15.52	1.69	0.07	15.22	0.16	1.61	0.06	7.89E-05	3.60E-03	1.51
8	0.03	8.50	-0.18	0.05	10.23	0.32	0.07	10.73	-0.72	0.46	0.05	-7.86E-06	5.13E-03	0.34
	0.02	10.12	-0.10	0.04	12.63	0.53	0.16	15.74	-7.38	1.31	0.04	-8.78E-05	4.05E-03	0.63
9	0.07	5.95	2.67	0.14	6.88	3.05	0.08	6.30	0.67	2.73	0.10	2.12E-04	1.22E-02	2.84
	0.06	7.79	1.77	0.10	9.13	1.99	0.07	8.56	0.55	1.85	0.08	1.32E-04	8.07E-03	1.91
10	0.052	21.85	1.53	0.069	27.53	2.09	0.05	24.90	0.53	1.66	0.060	9.10E-05	1.72E-03	1.78
	0.059	24.41	2.40	0.077	30.81	2.95	0.06	27.03	0.63	2.43	0.067	9.08E-05	1.72E-03	2.64

Table S2−2. Estimated parameter values of the photosynthetic light-response models using both the Differential Evolution and the Nelder-Mead algorithms.

Species	Exponential			Rectangular			Nonrectangular				Modified Rectangular			
	a	Amax	Rd	a	Amax	Rd	a	Amax	θ	Rd	a	β	γ	Rd
11	0.055	9.31	0.43	0.083	10.86	0.76	0.04	9.25	0.88	0.16	0.059	2.16E-04	3.78E-03	0.45
	0.047	6.55	0.69	0.072	7.44	0.88	0.03	6.04	0.99	0.23	0.043	3.37E-04	3.11E-03	0.58
12	0.046	15.71	0.42	0.063	19.38	0.90	0.06	18.86	0.19	0.81	0.064	-1.36E-05	3.41E-03	0.93
	0.052	20.69	0.79	0.070	25.90	1.39	0.06	24.82	0.27	1.21	0.066	3.98E-05	2.35E-03	1.30
13	0.07	54.08	3.87	0.09	74.42	5.42	0.08	71.41	0.18	4.99	0.08	2.89E-05	1.03E-03	5.05
	0.05	31.39	3.45	0.09	42.32	6.55	0.05	36.22	0.57	3.61	0.07	5.46E-05	1.46E-03	5.32
14	0.02	4.93	0.10	0.03	5.78	0.26	0.03	5.57	0.36	0.21	0.03	5.14E-05	4.52E-03	0.22
	0.02	5.74	-0.07	0.03	6.74	0.10	0.03	6.55	0.28	0.06	0.03	8.23E-05	3.48E-03	0.03
15	0.07	30.36	2.31	0.09	38.29	2.86	0.07	33.47	0.65	2.31	0.08	9.89E-05	1.52E-03	2.51
	0.10	40.56	3.08	0.12	50.87	3.60	0.08	42.67	0.79	2.74	0.10	1.19E-04	1.43E-03	3.13
16	0.10	25.36	-0.28	0.13	31.06	0.26	0.06	24.65	0.93	-1.18	0.09	2.79E-04	1.52E-03	-0.57
	0.09	18.60	-0.71	0.12	22.34	-0.18	0.07	18.90	0.85	-1.13	0.09	1.98E-04	2.85E-03	-0.67
17	0.047	9.33	2.22	0.070	10.90	2.57	0.05	10.15	0.57	2.32	0.063	5.57E-05	5.21E-03	2.47
	0.046	11.86	1.92	0.064	14.18	2.26	0.04	12.80	0.65	1.96	0.056	7.51E-05	3.40E-03	2.12
18	0.028	7.58	1.00	0.041	9.09	1.27	0.03	8.51	0.47	1.12	0.037	5.19E-05	3.70E-03	1.20
	0.018	4.79	0.93	0.027	5.74	1.13	0.02	5.61	0.20	1.09	0.025	4.46E-05	3.94E-03	1.08
19	0.06	13.37	0.81	0.08	15.82	1.28	0.06	14.96	0.46	1.04	0.08	3.09E-05	4.68E-03	1.20
	0.08	23.94	2.31	0.11	28.93	2.98	0.07	25.82	0.68	2.26	0.09	1.07E-04	2.40E-03	2.52
20	0.024	11.65	-0.35	0.034	14.80	0.04	0.05	17.32	-1.49	0.28	0.043	-1.43E-04	3.81E-03	0.28
	0.045	12.58	0.43	0.062	15.16	0.81	0.04	13.75	0.62	0.49	0.056	6.04E-05	3.28E-03	0.68
21	0.07	8.51	0.64	0.10	9.61	0.74	0.04	8.35	0.93	0.38	0.08	1.73E-04	6.29E-03	0.66
	0.05	16.68	0.40	0.07	21.11	0.63	0.04	16.83	0.87	0.10	0.05	1.96E-04	1.63E-03	0.35

Table S3-1. Times of failed convergence of the Levenberg-Marquardt in the simulation test.

Species	Exponential	Rectangular	Nonrectangular	Modified Rectangular
1	4	8	30	2
	6	8	30	5
2	13	1	19	18
	18	0	20	29
3	3	0	36	34
	5	0	38	31
4	3	0	25	23
	7	8	31	2
5	13	0	27	45
	2	0	27	30
6	4	0	25	42
	4	0	22	31
7	4	0	31	30
	9	6	41	6
8	6	0	34	25
	13	5	23	3
9	5	7	19	11
	2	0	28	36
10	6	9	25	5
	6	9	31	5

Table S3-2. Times of failed convergence of the Levenberg-Marquardt in the simulation test.

Species	Exponential	Rectangular	Nonrectangular	Modified Rectangular
11	7	11	22	3
	11	9	23	3
12	7	8	26	2
	4	9	24	2
13	50	47	51	54
	64	56	65	59
14	2	0	26	39
	2	0	27	33
15	4	0	50	21
	10	0	52	20
16	1	0	23	17
	1	1	25	19
17	6	8	21	0
	5	10	24	1
18	9	10	26	2
	11	11	28	2
19	2	0	28	22
	2	0	29	16
20	5	0	28	30
	6	10	30	3
21	8	0	30	27
	8	0	40	32
Total	358	251	1260	820

Table S4. List of the species used to test the performance of the new methods.

Species	Latin name	Family
1	*Spinacia oleracea* L.	*Amaranthaceae*
2	*Atriplex subspicata* Nutt. Rydb.	*Amaranthaceae*
3	*Impatiens balsamina* L.	*Balsaminaceae*
4	*Brassica rapa* var. *chinensis* (L.) Kitam.	*Brassicaceae*
5	*Arabidopsis thaliana* (Columbia gl1)	*Brassicaceae*
6	*Cucumis hystrix* Chakrav.	*Cucurbitaceae*
7	*Vaccinium corymbosum* L.	*Ericaceae*
8	*Quercus suber* L.	*Fagaceae*
9	*Lindera melissifolia* (Walter) Blume	*Lauraceae*
10	*Glycine max* (L.) Merr.	*Leguminosae*
11	*Trifolium repens* L.	*Leguminosae*
12	*Eucalyptus grandis* W. Hill	*Myrtaceae*
13	*Eucalyptus maculata* Hook.	*Myrtaceae*
14	*Pinus palustris* Mill.	*Pinaceae*
15	*Saccharum* spp.	*Poaceae*
16	*Secale cereale* L. cv Musketeer	*Poaceae*
17	*Rosa chinensis* Jacq.	*Rosaceae*
18	*Simmondsia chinensis* (Link) Schneider	*Simmondsiaceae*
19	*Nicotiana tabacum*L.	*Solanaceae*
20	*Camellia sinensis* (L.) Kuntze	*Theaceae*
21	*Vitis vinifera* L.	*Vitaceae*
